# Insects and Flowers

**Published:** 1885-04

**Authors:** 


### Insects and Flowers.

It has long been known that flowers were necessary to insects; but it is only within the last few years that it has been discovered that insects are quite as necessary to flowers. There are, however, but two or three tribes of insects whose visits are serviceable to flowers in the way of fertilization. The Lepidoptera or butterfly tribe are especially so, and the moths flying by night, and visiting such flowers as are only open at that time, are furnished with a trunk or proboscis which sucks up honey in its fluid state, and in seeking it the insect becomes covered with pollen which it transfers from flower to flower. In this way a single insect will fertilize many flowers. Besides being attracted by the color of flowers, insects seem capable of appreciating taste and smell, just as the higher animals do. What flowers are to insects, fruits are to birds and mammals. Both are colored, scented, sweet; but they have acquired their various allurements for the attraction of widely different creatures.--*Chamber's Journal.*

